# Supplementary material for: Joint trajectories of physical activity, health, and income before and after statutory retirement: A 22-year follow-up
Source: PLoS One. 2025 Jan 29;20(1):e0317010. doi: 10.1371/journal.pone.0317010 (PMC11778762; doi:10.1371/journal.pone.0317010)
Supplement: S1 File — (PDF) [file pone.0317010.s001.pdf]

Supporting information

S1 Table. Values of model selection criteria for different numbers of trajectory groups (Phases 1–5, 2000–2022, Helsinki Health Study).

S2 Table. Posterior probabilities for model containing four trajectory groups (Phases 1–5, 2000–2022, Helsinki Health Study).

S3 Table. Odds ratios (OR) for trajectory groups in multinomial logistic regression (with 95% confidence intervals) [Age and retirement age included]

S4 Table. Mean age of retirement

S5 Table. Variable distributions (%) in population lost to follow-up and study population (OR, 95% CI)

S1 Fig. Rootograms of posterior probabilities for selected four trajectory groups (Phases 1–5, 2000–2022, Helsinki Health Study).

S2 Fig. Plot of the selection criteria for best number of trajectory groups: Akaike Information Criterion (AIC), Bayesian Information Criterion (BIC) and Integrated Complete Likelihood (ICL) (Phases 1–5, 2000–2022, Helsinki Health Study)

S3 Fig. Joint development of leisure-time physical activity (metabolic equivalent, MET, hours), general health perceptions (score 0–100), and household income (€) 10 years before and after statutory retirement (x-axis) among Helsinki Health Study participants 2000–2022 (n=5209). Joint Group-based Trajectory Modeling with five groups. Group 1 (18.7%), Group 2 (10.4%), Group 3 (21.4%), Group 4 (21.5%), Group 5 (27.9%)

S4 Fig. Rootograms of posterior probabilities for five trajectory groups (Phases 1–5, 2000–2022, Helsinki Health Study)

S5 Fig. Joint development of leisure-time physical activity (metabolic equivalent, MET, hours), general health perceptions (score 0–100), and household income (€) 10 years before and after statutory retirement (x-axis) among Helsinki Health Study participants 2000–2022 (n=5209). Joint Group-based Trajectory Modeling with six groups. Group 1 (16.4%), Group 2 (16.2%), Group 3 (24.5%), Group 4 (17.4%), Group 5 (16.3%), Group 6 (9.2%)

S6 Fig. Rootograms of posterior probabilities for six trajectory groups (Phases 1–5, 2000–2022, Helsinki Health Study)

S1 File. Additional methods Supplement: Survey questions used in the study

S1 Table. Values of model selection criteria for different numbers of trajectory groups (Phases 1–5, 2000–2022, Helsinki Health Study)

Number of trajectory groups	Log-likelihood function (logLik)	Akaike Information Criteria (AIC)	Bayesian Information Criteria (BIC)	Integrated Complete Likelihood (ICL)
1	-392739.0	785501.9	785598.6	785598.6
2	-385441.1	770932.1	771133.6	771788.5
3	-382090.8	764257.6	764563.8	765497.8
4	-379792.3	759686.7	760097.7	761194.8
5	-378445.4	757018.8	757534.5	758818.0
6	-377380.0	754914.0	755534.5	756996.9
7	-376576.2	753332.3	754057.6	755692.3
8	-375962.0	752130.1	752960.1	754677.3
9	-375409.6	751051.2	751986.0	753784.7
10	-374918.0	750094.1	751133.6	753019.6

S2 Table. Posterior probabilities for model containing four trajectory groups (Phases 1–5, 2000–2022, Helsinki Health Study)

	Group 1 posterior probabilities	Group 2 posterior probabilities	Group 3 posterior probabilities	Group 4 posterior probabilities
Group 1 members	**0.924**	0.028	0.021	0.027
Group 2 members	0.037	**0.903**	0.041	0.019
Group 3 members	0.026	0.057	**0.917**	0.000
Group 4 members	0.040	0.035	0.000	**0.925**

S3 Table. Odds ratios (OR) for trajectory groups in multinomial logistic regression (with 95% confidence intervals) [Age and retirement age included]

	Group 1 [n = 1177]	Group 2 [n = 1783]	Group 3 [n = 639]	Group 4 [n = 1610]
Gender				
Men (ref =1)	-	-	-	-
Women	1.61 (1.41 | 1.84)	2.37 (2.10 | 2.67)	-	1.69 (1.52 | 1.89)
Marital status				
Co-habiting/married (ref =1)	-	-	-	-
Never married/divorced/widowed	2.08 (1.86 | 2.34)	2.93 (2.64 | 3.25)	-	0.46 (0.41 | 0.52)
Education				
Higher education (ref =1)	-	-	-	-
Upper secondary	1.42 (1.24 | 1.63)	1.80 (1.59 | 2.02)	-	0.63 (0.56 | 0.70)
Lower secondary	2.20 (1.87 | 2.59)	3.30 (2.85 | 3.81)	-	0.41 (0.35 | 0.47)
Low-level	3.04 (2.58 | 3.58)	3.85 (3.32 | 4.47)	-	0.39 (0.33 | 0.45)
Average hours of sleep				
7-8 hours (ref =1)	-	-	-	-
Less than 7 hours	1.57 (1.38 | 1.78)	1.17 (1.04 | 1.32)	-	1.05 (0.94 | 1.18)
9 hours or more	1.20 (0.97 | 1.49)	1.15 (0.94 | 1.40)	-	1.02 (0.84 | 1.24)
Obesity				
Normal weight (ref =1)	-	-	-	-
Overweight	3.37 (3.00 | 3.79)	1.98 (1.79 | 2.19)	-	2.17 (1.96 | 2.40)
Obesity	15.20 (12.55 | 18.41)	3.79 (3.13 | 4.57)	-	5.20 (4.32 | 6.26)
Smoking				
No smoking (ref =1)	-	-	-	-
Current smoking	2.72 (2.29 | 3.24)	1.83 (1.55 | 2.15)	-	1.45 (1.22 | 1.71)
Past smoking	0.77 (0.68 | 0.86)	0.83 (0.75 | 0.91)		0.93 (0.84 | 1.03)
Binge drinking				
No (ref =1)	-	-	-	-
Binge drinking (once a week or more)	1.49 (1.23 | 1.80)	1.09 (0.91 | 1.31)	-	1.24 (1.04 | 1.47)
Number of physician-diagnozed chronic diseases and mental disorders				
0 (ref =1)	-	-	-	-
1	1.82 (1.60 | 2.08)	1.20 (1.08 | 1.34)	-	1.38 (1.24 | 1.54)
2	3.32 (2.84 | 3.87)	1.44 (1.25 | 1.65)	-	1.69 (1.48 | 1.93)
3 or more	9.82 (8.09 | 11.91)	2.22 (1.84 | 2.67)	-	2.51 (2.09 | 3.02)
Physically strenuous work				
No (ref =1)	-	-	-	-
Low level of physically strenuous work	1.59 (1.39 | 1.81)	1.55 (1.38 | 1.74)	-	1.01 (0.91 | 1.12)
High level of physically strenuous work	2.12 (1.82 | 2.47)	1.80 (1.57 | 2.06)	-	0.74 (0.65 | 0.85)
Age				
54 or younger (ref =1)	-	-	-	-
54–59	0.96 (0.81 | 1.15)	1.13 (0.96 | 1.31)	-	1.06 (0.91 | 1.23)
60 or older	1.01 (0.87 | 1.16)	1.25 (1.10 | 1.42)	-	1.13 (1.00 | 1.28)
Age of retirement				
59 or younger (ref =1)	-	-	-	-
60–64	1.19 (0.90 | 1.58)	1.10 (0.85 | 1.41)	-	1.72 (1.30 | 2.28)
65 or older	1.11 (0.84 | 1.48)	1.06 (0.82 | 1.37)	-	1.57 (1.18 | 2.01)

S4 Table. Mean age of retirement (sd., standard deviation)

Group 1 [n = 1177]	Group 2 [n = 1783]	Group 3 [n = 639]	Group 4 [n = 1610]	All [n = 5209]
mean 64.06. sd. 3.13	mean 63.96. sd. 2.95	mean 63.89. sd. 2.68	mean 63.91. sd. 2.50	mean 63.96, sd. 2.83

S5 Table. Variable distributions (%) in population lost to follow-up and study population (OR, 95% CI)

Phase 1 variables	Lost to follow-up [n = 368]	Study population [n = 5209]	p-value (chi-squared)
Gender			0.027
Men	24.5	19.6	
Women	75.5	80.4	
Age			1.93E-15
Under 55	32.9	49.7	
55-59	35.3	33.9	
60 or older	31.8	16.3	
Marital status*			0.045
Co-habiting/married	64.9	70.7	
Never married/divorced/widowed	33.4	28.7	
Education*			6.76E-08
Higher education	19.8	28.0	
Upper secondary	21.7	29.3	
Lower secondary	23.1	20.6	
Low-level	32.6	21.4	
Average hours of sleep*			0.006
7–8 hours	63.9	71.8	
Less than 7 hours	31.5	24.7	
9 hours or more	4.3	3.5	
Obesity			
Normal weight	44.0	49.3	0.053
Overweight	37.5	36.3	
Obesity	18.5	14.4	
Smoking*			0.046
No smoking	50.5	54.0	
Past smoking	22.6	25.2	
Current smoking	25.3	20.1	
Binge drinking*			
No	83.7	88.3	0.035
Binge drinking (once a week or more)	12.8	9.4	
Number of physician-diagnozed chronic diseases and mental disorders			0.723
0	47.3	48.3	
1	33.4	31.0	
2	11.7	13.2	
3 or more	7.6	7.5	
Physically strenuous work*			4.14E-04
No	44.3	43.8	
Low level of physically strenuous work	38.9	33.3	
High level of physically strenuous work	12.8	21.4	
Leisure-time physical activity			0.008
Mean MET-hours	27	29	
Perceived general health, mean score (range 0–100)	63	67	0.41
Household income, mean €	1166	1247	6.36E-05

*some variables have a small number (typically 1–2%) of missing values and thus all rows do not amount to 100%. Missing values were excluded from the models or replaced by mean values (body mass index and sleep duration). More details are given in the main text.

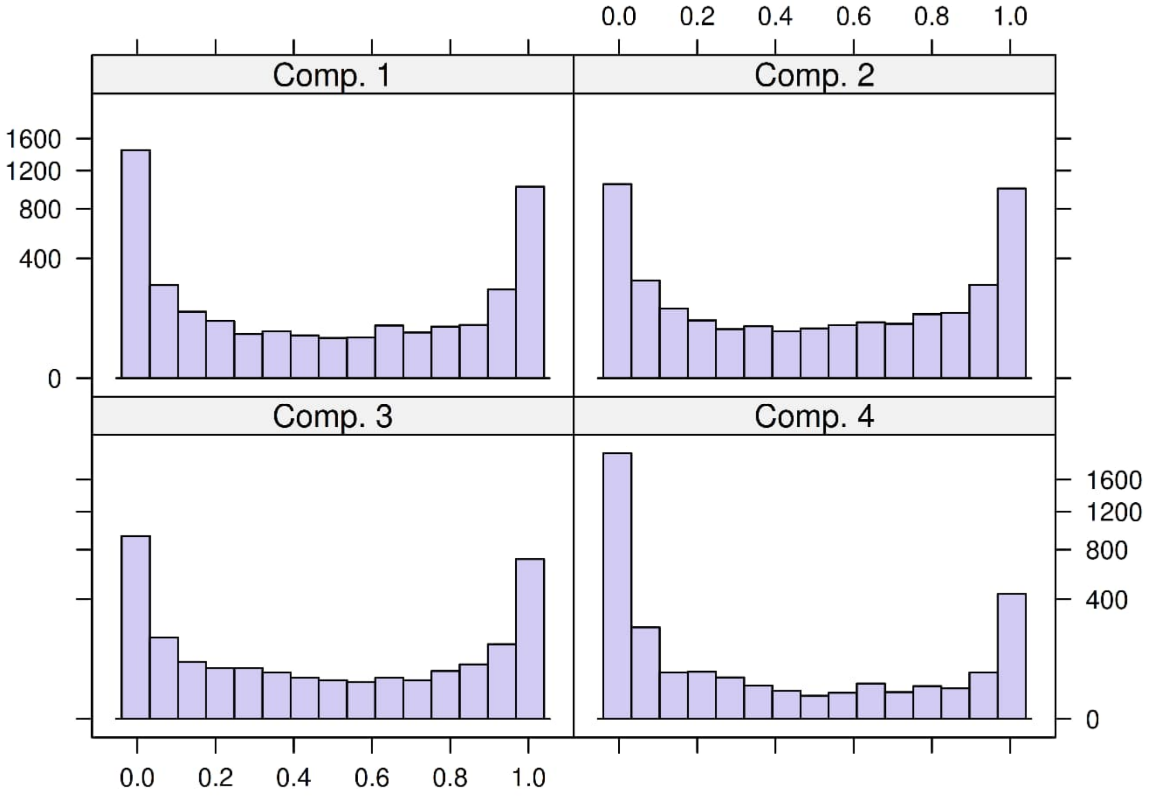

S1 Fig. Rootograms of posterior probabilities for selected four trajectory groups (Phases 1–5, 2000–2022, Helsinki Health Study)

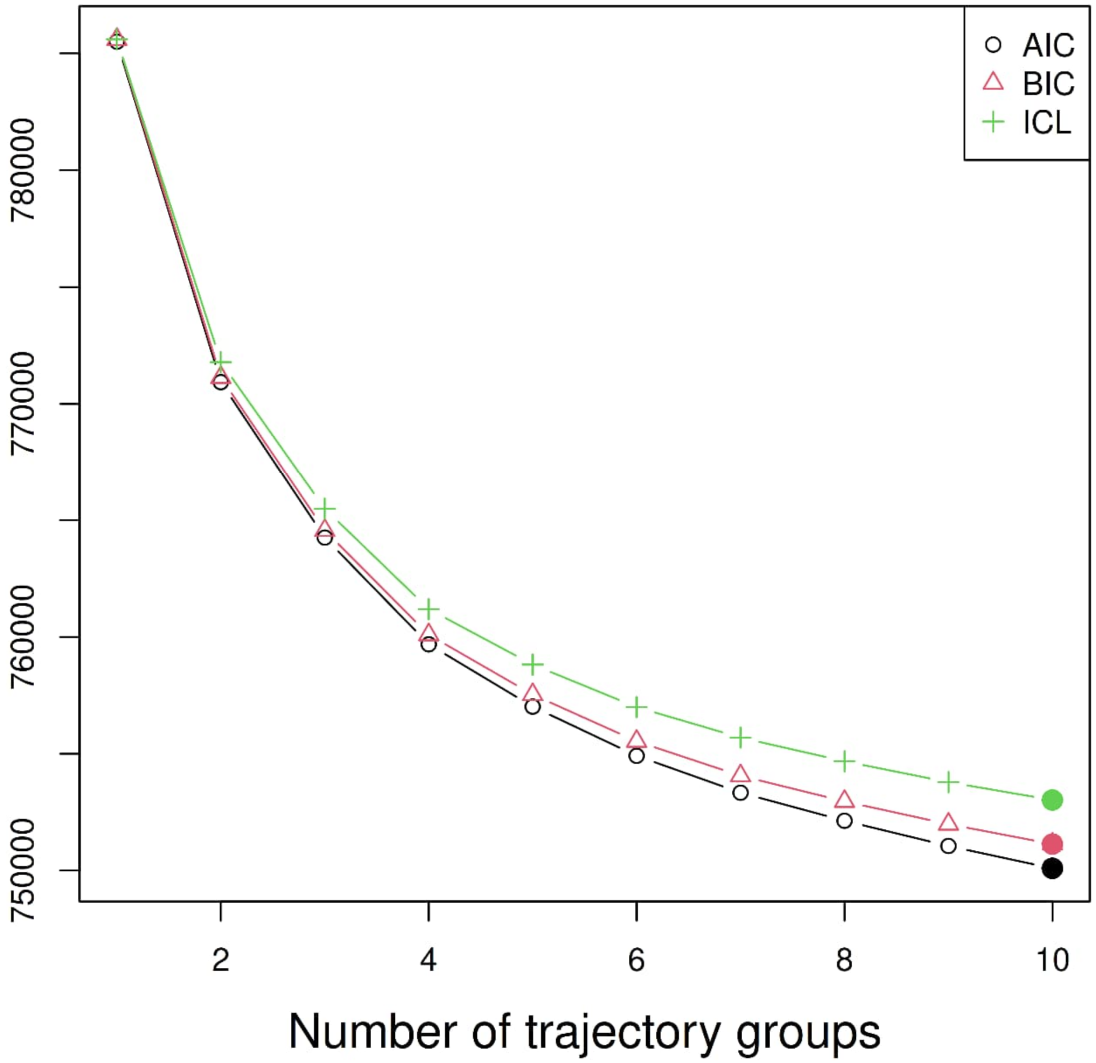

S2 Fig. Plot of the selection criteria for best number of trajectory groups: Akaike Information Criterion (AIC), Bayesian Information Criterion (BIC) and Integrated Complete Likelihood (ICL) (Phases 1–5, 2000–2022, Helsinki Health Study)

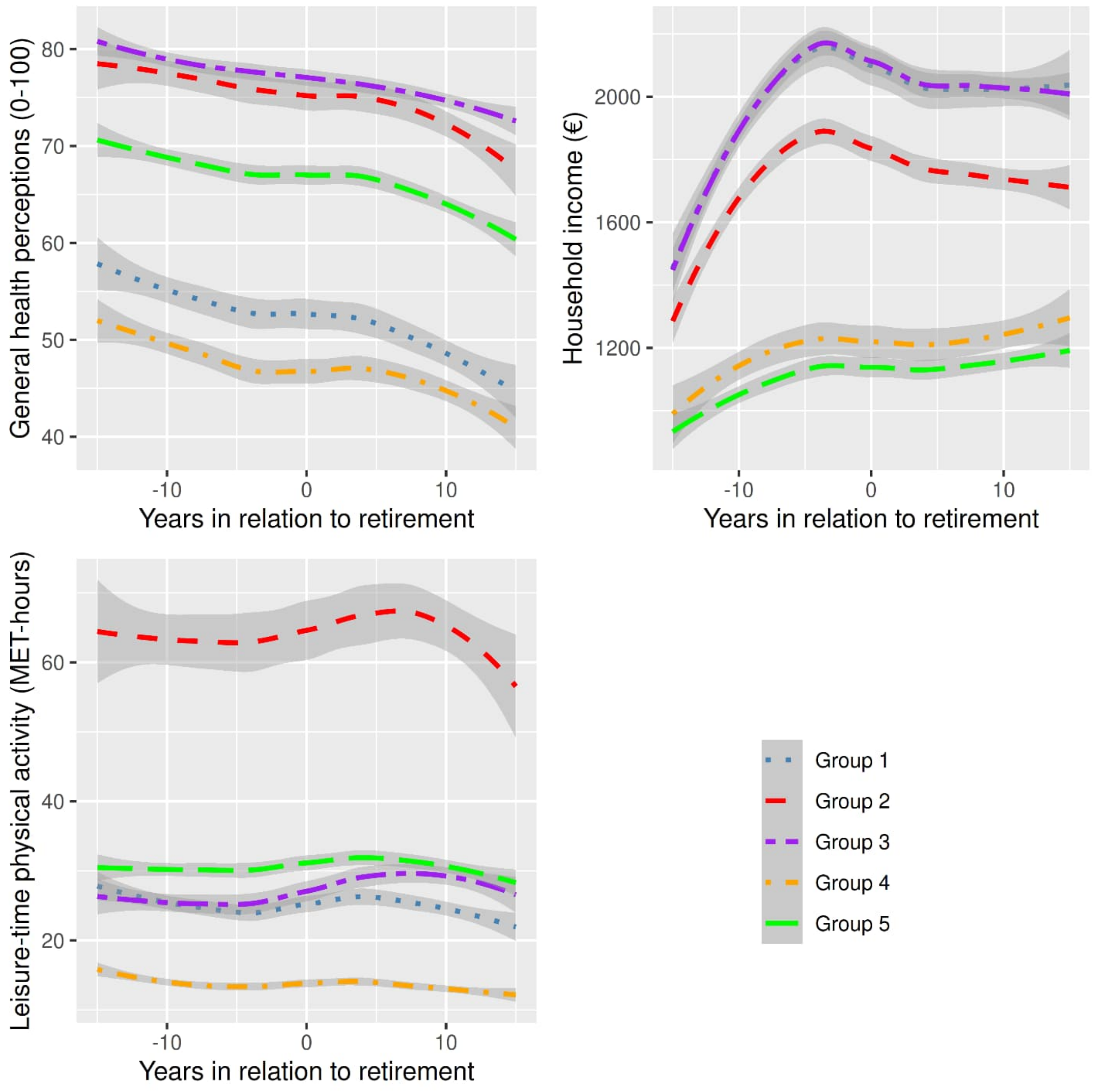

S3 Fig. Joint development of leisure-time physical activity (metabolic equivalent, MET, hours), general health perceptions (score 0–100), and household income (€) 10 years before and after statutory retirement (x-axis) among Helsinki Health Study participants 2000–2022 (n=5209). Joint Group-based Trajectory Modeling with five groups. Group 1 (18.7%), Group 2 (10.4%), Group 3 (21.4%), Group 4 (21.5%), Group 5 (27.9%)

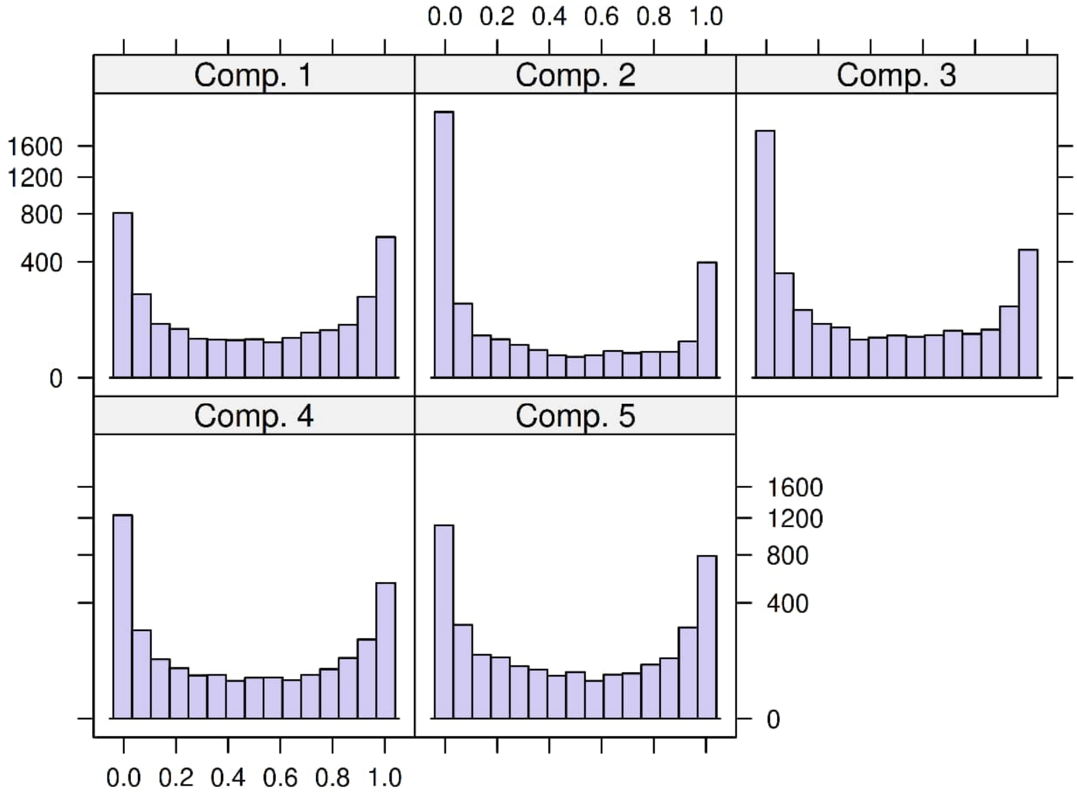

S4 Fig. Rootograms of posterior probabilities for five trajectory groups (Phases 1–5, 2000–2022, Helsinki Health Study)

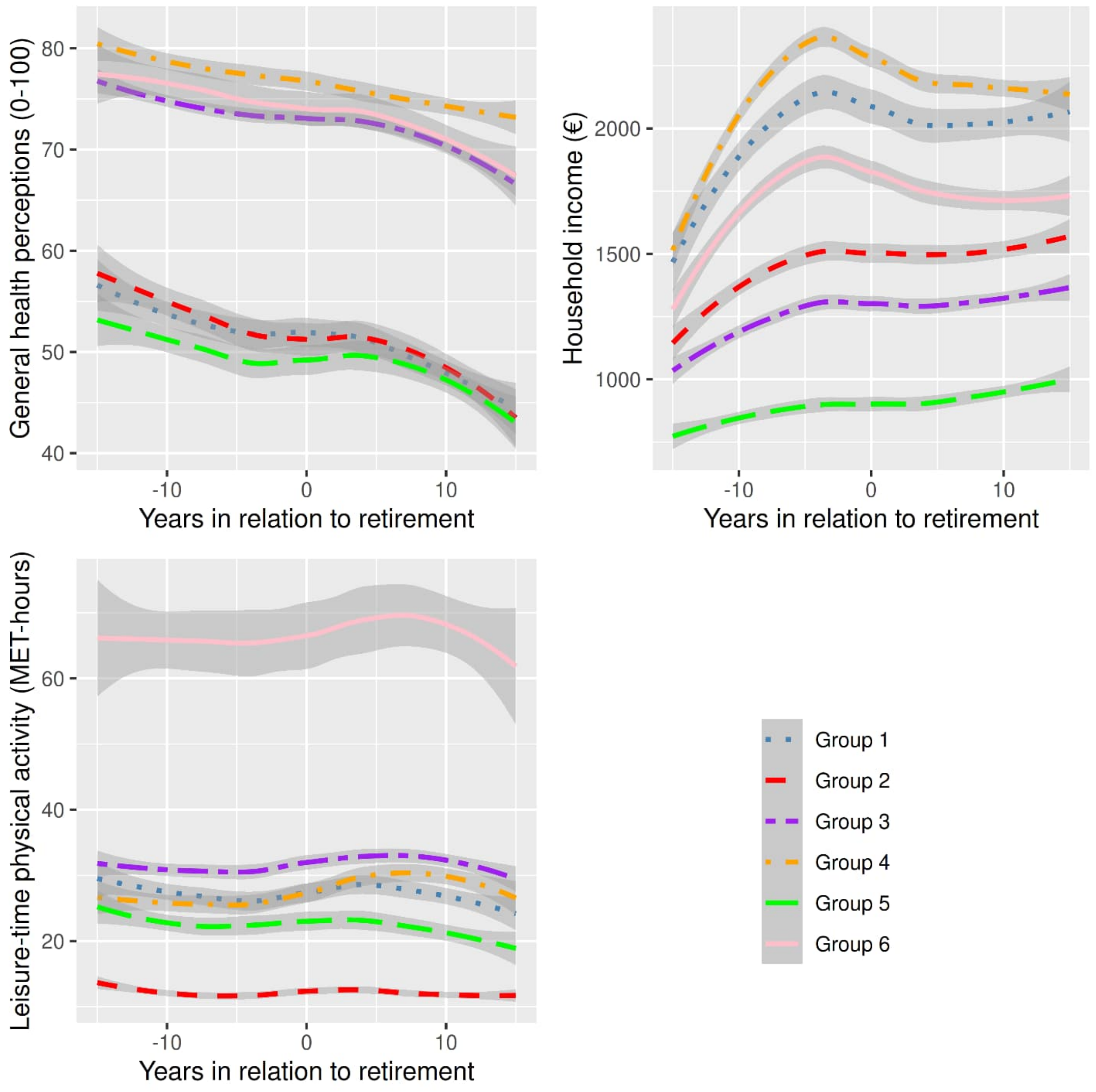

S5 Fig. Joint development of leisure-time physical activity (metabolic equivalent, MET, hours), general health perceptions (score 0–100), and household income (€) 10 years before and after statutory retirement (x-axis) among Helsinki Health Study participants 2000–2022 (n=5209). Joint Group-based Trajectory Modeling with six groups. Group 1 (16.4%), Group 2 (16.2%), Group 3 (24.5%), Group 4 (17.4%), Group 5 (16.3%), Group 6 (9.2%)

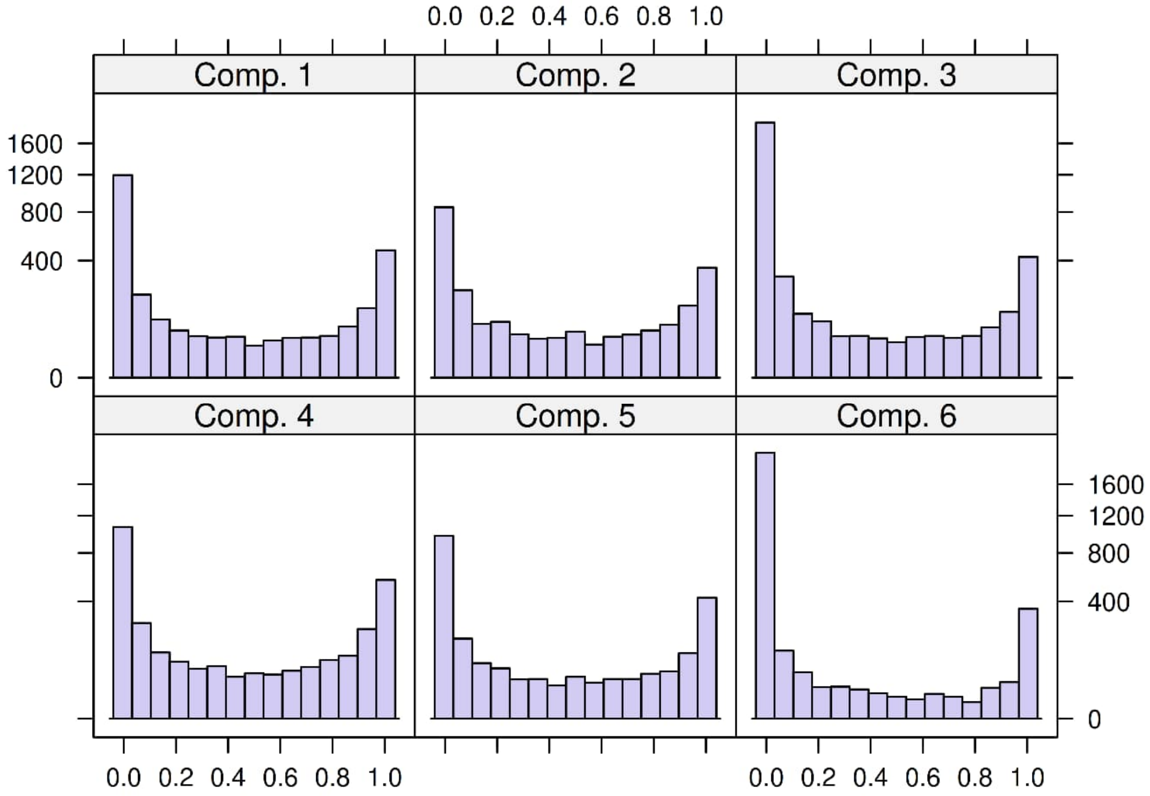

S6 Fig. Rootograms of posterior probabilities for six trajectory groups (Phases 1–5, 2000–2022, Helsinki Health Study)

S1 File. Additional methods Supplement: Survey questions used in the study

*NB the survey was conducted in Finnish and Swedish only. Thus, the items in this supplement are translations to support the study.

1. What is your sex?
   ☐ male
   ☐ female

2. a. Are you currently mainly… Please tick all that apply.
   ☐ in full-time work
   ☐ in part-time work
   ☐ on part-time pension or partial early old-age pension
   ☐ on old-age pension
   ☐ on a long-term (over 6 months) sick leave
   ☐ on full or partial disability pension
   ☐ other, please specify: ____

b. If you are currently on pension, please enter the type and start date of the current pension.
   ☐ not on pension (please move to the next question)
   ☐ old-age pension, starting from:
   month _____ year _____
   ☐ partial early old-age pension (25% or 50%), starting from:
   month _____ year _____
   ☐ full disability pension (granted either until further notice or temporarily, in which case the disability pension is called cash rehabilitation benefit), starting from:
   month _____year ______
   ☐ partial disability pension (granted either until further notice or temporarily, in which case the disability pension is called partial cash rehabilitation benefit), starting from:
   month _____ year ______
   ☐ other pension, starting from:
   month _____ year ______

c. If you are currently on old-age pension, did you transfer to this pension from another pension? (ref. self-translated by Emmi and same as previous questions)
   ☐ no other previous pension
   ☐ partial early old-age pension (25% or 50%), starting from:
   month _____ year _____
   ☐ partial or full disability pension, starting from:
   month _____ year _____
   ☐ other pension, please specify: ____
   starting from: month _____ year _____

3. What is your marital status?
   - ☐ single (never married)
   - ☐ cohabiting
   - ☐ married or in a registered partnership
   - ☐ separated or divorced
   - ☐ widowed

4.
   a. In addition to you, does a spouse or a cohabiting partner live in your household?
   - ☐ yes
   - ☐ no

   b. How many children aged 0-18 live in your household?

   ☐ 0 ☐ 1 ☐ 2 ☐ 3 ☐ 4 ☐ 5+

   c. How many other adults live in your household excluding your spouse?

   ☐ 0 ☐ 1 ☐ 2 ☐ 3 ☐ 4 ☐ 5+

5. In which of the following income categories does your household belong? Estimate the combined income of all members of your household, minus taxes and including any housing benefit or similar transfers of income, in a typical month.

☐ less than 1200 euros	☐ 1200–1499 euros	☐ 1500–1699 euros
☐ 1700–1899 euros	☐ 1900–2199 euros	☐ 2200–2499 euros
☐ 2500–2999 euros	☐ 3000–3999 euros	☐ more than 4000 euros

HEALTH

6. Has a doctor ever diagnosed you with any of the following disorders?

	Yes	No
Gout, osteoporosis, osteoarthritis or rheumatoid arthritis	☐	☐
High blood pressure (hypertension)	☐	☐
High blood cholesterol	☐	☐
Coronary artery disease or other cardiovascular disease	☐	☐
Cerebrovascular disease	☐	☐
Chronic obstructive pulmonary disease (COPD)	☐	☐
Asthma	☐	☐
Sleep apnoea	☐	☐
Depression	☐	☐
Anxiety disorder	☐	☐
Other mental disorder	☐	☐
Migraine	☐	☐
Parkinson’s disease	☐	☐
Memory disease (Alzheimer's disease or other dementia)	☐	☐
Thyroid disease	☐	☐
Diabetes	☐	☐
Long-term skin disease	☐	☐
Celiac disease	☐	☐
Irritable Bowel Syndrome (IBS)	☐	☐
Cancer	☐	☐

7. How many hours a day do you sleep on average on weekdays?
   - ☐ 5 hours or less
   - ☐ 6 hours
   - ☐ 7 hours
   - ☐ 8 hours
   - ☐ 9 hours
   - ☐ 10 hours or more

General health perceptions

8. In general, would you say your health is:
   - ☐ excellent
   - ☐ very good
   - ☐ good
   - ☐ fair
   - ☐ poor

9.

	Definitely true	Mostly true	Don’t know	Mostly false	Definitely false
I seem to get sick a little easier than other people	☐	☐	☐	☐	☐
I am as healthy as anybody I know	☐	☐	☐	☐	☐
I expect my health to get worse	☐	☐	☐	☐	☐
My health is excellent	☐	☐	☐	☐	☐

HEIGHT AND WEIGHT

10. How tall are you?
    ______ cm (only indicate full centimetres)

11. How much do you weigh?
    ______ kg (only indicate full kilograms)

HEALTH BEHAVIOUR

12. Do you currently smoke cigarettes, cigars or a pipe regularly?
    ☐ yes
    ☐ no (if no, please move to question 32)

13. How much, on average, do you consume the following alcoholic beverages?
    a. Beer or cider
    ☐ none
    ☐ less than one bottle a week
    ☐ 1–4 bottles a week
    ☐ 5–12 bottles a week
    ☐ 13–24 bottles a week
    ☐ 25–47 bottles a week
    ☐ 48 bottles or more a week

    b. Wine or equivalent alcoholic beverage
    ☐ none
    ☐ less than a glass a week
    ☐ 1–4 glasses a week
    ☐ 1–2.5 bottles a week
    ☐ 3–4.5 bottles a week
    ☐ 5–9 bottles a week
    ☐ 10 bottles or more a week

    c. Spirits
    ☐ none
    ☐ less than half a bottle a month
    ☐ 0.5–1.5 bottles a month
    ☐ 2–3.5 bottles a month
    ☐ 4–9 bottles a month
    ☐ 10–19 bottles a month
    ☐ 20 bottles or more a month

14. The next question concerns situations in which you drink six or more servings of alcoholic beverages at one sitting. Six or more servings is equivalent to at least:

    - 4 pints (0.5 l each) of medium-strength beer/mild cider or
    - 3 pints (0.5 l each) of strong beer/strong cider or
    - one bottle (0.75 l) of mild wine (12%) or

- 6 restaurant servings (4 cl each) of spirits

How often do you drink six or more servings of alcoholic beverages at one sitting?

☐ never
☐ less than once a month
☐ once a month
☐ once a week
☐ a few times a week
☐ every day or almost every day

15. Next, we will be asking about physical activity during your leisure and commuting time over the past 12 months. We have divided physical activities in four levels of exertion. First, estimate the exertion level of the physical activities you are engaged in. Then, estimate how often you engage in a physical activity equivalent to each level of exertion during one week.

Strenuousness of exercise:	Not at all	Total under 0.5 hours a week	Total 0.5-1 hour a week	Total 2-3 hours a week	Total 4 hours or more a week
Equivalent to walking	☐	☐	☐	☐	☐
Equivalent to brisk walking	☐	☐	☐	☐	☐
Equivalent to light running (jogging)	☐	☐	☐	☐	☐
Equivalent to brisk running	☐	☐	☐	☐	☐

WORK, WORKING ENVIRONMENT AND COMMUNITY

16. What is your current occupation? ________________________________

17. What is your job like?

	Very light	Fairly light	Quite strenuous	Very strenuous
Physically	☐	☐	☐	☐
Mentally	☐	☐	☐	☐
