# Supplementary material for: Joint trajectories of physical activity, health, and income before and after statutory retirement: A 22-year follow-up
Source: PLoS One. 2025 Jan 29;20(1):e0317010. doi: 10.1371/journal.pone.0317010 (PMC11778762; doi:10.1371/journal.pone.0317010)
Supplement: Attachment — Submitted filename: Response to Reviewers.docx [file pone.0317010.s002.docx]

Christoph Strumann
Academic Editor
PLOS ONE

Journal requirements:

When submitting your revision, we need you to address these additional requirements.

1. Please ensure that your manuscript meets PLOS ONE's style requirements, including those for file naming. The PLOS ONE style templates can be found at 
<https://journals.plos.org/plosone/s/file?id=wjVg/PLOSOne_formatting_sample_main_body.pdf> and 
<https://journals.plos.org/plosone/s/file?id=ba62/PLOSOne_formatting_sample_title_authors_affiliations.pdf>

**RESPONSE: We ensure that the manuscript meets the PLOS ONE’S style requirements, as requested.**

2. Thank you for stating the following financial disclosure: 
 [This study is supported by the Research Council of Finland (Grant #330527) and OR is supported by the Ministry of Education and Culture, Finland (Grant #OKM/76/626/2023) and by the Juho Vainio Foundation (Grant #202300041)].  
Please state what role the funders took in the study.  If the funders had no role, please state: ""The funders had no role in study design, data collection and analysis, decision to publish, or preparation of the manuscript."" 
If this statement is not correct you must amend it as needed. 
Please include this amended Role of Funder statement in your cover letter; we will change the online submission form on your behalf.

**RESPONSE: Thank you. We confirm that the statement is correct. The funders had no role in this study, and we have included the statement, as requested.**

3. We note that you have indicated that there are restrictions to data sharing for this study. PLOS only allows data to be available upon request if there are legal or ethical restrictions on sharing data publicly. For more information on unacceptable data access restrictions, please see <http://journals.plos.org/plosone/s/data-availability#loc-unacceptable-data-access-restrictions>.

Before we proceed with your manuscript, please address the following prompts:

a) If there are ethical or legal restrictions on sharing a de-identified data set, please explain them in detail (e.g., data contain potentially identifying or sensitive patient information, data are owned by a third-party organization, etc.) and who has imposed them (e.g., a Research Ethics Committee or Institutional Review Board, etc.). Please also provide contact information for a data access committee, ethics committee, or other institutional body to which data requests may be sent.

b) If there are no restrictions, please upload the minimal anonymized data set necessary to replicate your study findings to a stable, public repository and provide us with the relevant URLs, DOIs, or accession numbers. For a list of recommended repositories, please see
<https://journals.plos.org/plosone/s/recommended-repositories>. You also have the option of uploading the data as Supporting Information files, but we would recommend depositing data directly to a data repository if possible.

We will update your Data Availability statement on your behalf to reflect the information you provide.

**RESPONSE: These data are highly sensitive, even after pseudonymization, and we are not permitted by law to share the data (General Data Protection Regulations, GDPR). More details on the Act are available at:** [**https://fra.europa.eu/en/law-reference/personal-data-act-5231999**](https://fra.europa.eu/en/law-reference/personal-data-act-5231999) **(Personal Data Act (523/1999)). These data can only be used by the research group, on a secure server, based on strict confidentiality. This has also been informed to all study participants. The University of Helsinki Information Security Manager Kenneth Kahri (**[**kenneth.kahri@helsinki.fi**](mailto:kenneth.kahri@helsinki.fi)**) has assessed the data sensitivity and based on the data, the decision was that these data can only be stored at a university server, confirmed to be sufficiently secure to manage any risks.**

**Our data protection document available at our webpage includes all the details:** [**https://www.helsinki.fi/en/researchgroups/helsinki-health-study/data-protection-statement**](https://www.helsinki.fi/en/researchgroups/helsinki-health-study/data-protection-statement)**.**

**The research ethics committee of the Faculty of Medicine has given a positive statement about the study. Its contact details are found at:** [**https://www.helsinki.fi/en/research/services-researchers/ethical-review-research/medical-research**](https://www.helsinki.fi/en/research/services-researchers/ethical-review-research/medical-research)

**Further details about legal aspects can be asked from the university lawyers** [**tutkimuksenjuristit@helsinki.fi**](mailto:tutkimuksenjuristit@helsinki.fi) **and about the data management from the data support team:** [**datasupport@helsinki.fi**](mailto:datasupport@helsinki.fi)**. All requests about the original data can be sent to:** [**kttl-hhs@helsinki.fi**](mailto:kttl-hhs@helsinki.fi)**.**

[Note: HTML markup is below. Please do not edit.]

**Reviewers' comments:**
Reviewer #1

In this survey-based study over a 22-year follow-up among public health workers, the authors investigated the joint trajectories of family income, leisure time, physical activity, and health. They have identified four trajectories and concluded that physical activity and health trajectories are not strictly linked to income trajectories and that even individuals with low-income trajectories can follow high physical activity and good health trajectories, suggesting that income and health might be less strongly correlated in the Finnish setting. The manuscript is well-written, and the method is adequate. However, there are some limitations of the study that the authors should discuss further.

Comments:

\# Why was sleeping categorized as such? According to public health recommendations and sleep research, 7-9 hours of sleep is considered healthy and is associated with the lowest mortality.

**RESPONSE: We appreciate this comment. Our sleep measure has a minor role in this study. It is but a covariate and it is rather crude. Unfortunately, sleep duration is asked to be reported only in full hours. For meaningful distribution, and to be able to distinguish between short, average, and long sleepers, we initially chose those responding 7 hours as a reference group (average sleep), while those sleeping less (6 hours or 5 hours or less) as short sleepers and those sleeping more than 7 hours (8 hours, 9 hours, or 10 hours or more) as long sleepers. A vast majority slept 7 or 8 hours (72.4%). For older adults, recommendations is 7–8 hours (**[**https://www.sciencedirect.com/science/article/abs/pii/S2352721815000157**](https://www.sciencedirect.com/science/article/abs/pii/S2352721815000157) **& http://dx.doi.org/10.1016/j.sleh.2015.10.004) and for young adults 7–9 hours. Additionally, if we used 7–9 hours as a reference, almost all participants would be placed in this group (75.9%). Almost nobody reported sleeping 10 hours or more (0.2%, 10 participants), and thus we would not be able to study long sleep at all. In sleep research, 7 and 8 hours are often combined, or even 6–8 hours to reflect average sleep, but as this cohort was employed at inclusion, it was extremely rare to report very short or long sleep. To have meaningful category sizes, and to somehow (with limitations), distinguish between short, average, and long sleepers, we thus chose this classification. If this was a sleep study, we fully agree we would need more detailed measures, or if we were interested in individual level sleep. However, for a covariate, and epidemiological purposes, we trust this variable works as a proxy for sleep quantity. We have amended the revised Methods to be clearer in describing these measures, and all our covariates and outcomes (following other comments). We now use 7–8 hours as a reference, to better meet with the recommendations.**

**We have clarified the methods and updated the classification following this comment (please see page 9).**

#The description of the result of the GBTM would fit better in the Results section.

**RESPONSE: Thank you for this comment. We are a bit unsure what it means, as all the results of the model are in the Results section. The statistical analysis and model selection criteria are described in the Methods section. It is how these model selection criteria are usually reported in previous literature, and it would feel somewhat strange to report statistical parts as results. We have thus retained the original place, as most of the description is technical and justifies the selected model. Description for other models tested would be confusing as results and it is difficult to separate statistical parts for the selected and other models.**

#Can the authors reason why they did not choose the 5 or 6 trajectory solutions, as these still resulted in a larger drop in AIC and BIC and have the lowest log-likelihood function?

**RESPONSE: It is true that often the models with more classes may appear superior if one only examines some statistical criteria such as AIC and BIC. Statistical criteria often tend to suggest more complex models with a larger number of classes. However, in trajectory analyses, there are many criteria guiding the selection of the best modes. A key one is related to class sizes: they have to be meaningful, as if the classes are really small, there is little population level relevance, and it is difficult to conduct any further analyses such as study determinants of trajectory membership. Additionally, the classes have to visibly distinct, which sometimes is not that obvious, even if some of the statistical criteria suggest those more complex solutions. Finally, we have to consider average posterior probabilities (APPs) and misclassification. Thus, there must be a meaningful story, and large enough classes (typically at least 100 individuals, or 5% or 10% class). AIC and BIC alone are unlikely to be sufficient in selecting the best model.**

**In our study, statistically, we might have chosen 5 or 6 class model even, but these did not add new information and all the groups were not as clearly distinct. In other words, when the number of classes increases, some classes are no longer clearly distinct as in our selected 4 class model. Some categories are also small, and it is difficult to examine associations, or provide population level messages.**

**We regret that our initial appendices did not clearly illustrate all these criteria for more complex models, or provided sufficient explanation for interpretation of the patterns. We have clarified the Methods section to better justify the 4-class solution and have included a more detailed Supplementary file (Fig S3) and examples of 5 and 6 class models in our supplementary material.**

#It seems that education is still a strong indicator of both income and health-related trajectories, while income is not correlated that closely with health-related trajectories. This is in line with the results of studies investigating different socioeconomic measures in relation to health outcomes, which I think would be useful to discuss.

**RESPONSE: We agree that education and income are not interchangeable and have different associations with health. It is true that education correlated with income and has clear and consistent associations with health outcomes, as mentioned in the manuscript. We have amended the revised manuscript to better highlight this. It is of note though that income is an outcome in the model while education is a from early adulthood, and asked to be reported at Phase 1 only, and it is only used in the multinomial models. As can be seen in the Figure, income changes over time and there are distinct groups among the participants. Education was included as a covariate in the model where income is one of the outcomes. Thus, one has to consider that education (covariate) and income (outcome) have very different roles in this study, and in the model with education, there is always income as an outcome, with health-related outcomes.**

\# “While high levels of physical activity are consistently linked with better health, this study also suggests that recommended level of physical activity is likely sufficient to maintain health in aging and older adults.” This is an overstatement and suggests causality, which was not possible to investigate with the chosen study design and method.

**RESPONSE: We fully agree we cannot suggest causality in this design. We have revised the section to be more cautious and in line with the study design, as suggested. Our intention is not to suggest causality. Joint group-based trajectory model is descriptive and only approximates true developmental patterns. It is, however, able to identify latent groups in the data and show if the chosen outcomes jointly co-occur. For example, the model could find people with declining health after they report a change in income or physical activity, if there was such a group in the data.**

#One of the largest limitations of this study is the possible selection bias due to selective dropout during the follow-up. Therefore, it would be important to present supportive data that attrition was not selective. I would suggest comparing the characteristics of those who dropped out during the follow-up vs those included in the study to have a clearer picture of this type of bias.

**RESPONSE: Thank you for this suggestion. This cohort actually has exceptionally high response rate throughout the follow-up. Thus, attrition is not a major issue, and our model can also use the data from the timepoints available. Different follow-up time does not affect this model. This choice avoids redundant selection as each participant could contribute to the trajectories even if they did not have all 5 time points (a vast majority though did have 4–5 time points and only 0.1% had 2 time points, they are now excluded). However, we agree that attrition can still be selective, and one could assume that those with the poorest health, low physical activity and low income are over-represented among those lost to follow-up. If they still contributed to at least 3 phases, their responses could be used. We have studied loss to follow-up by the key variables of interest (gender, age, marital status, education, physically strenuous work, smoking, binge drinking, sleep duration, obesity, physician diagnosed diseases, income, leisure-time physical activity, and general health) and the results are included as a new Supplement Table S5 and briefly covered in the revised Discussion (please see page 23). These covariates were taken from Phase 1 and then we examined loss to follow-up as an outcome (lost to follow-up =1, included in this study =0). Those who were working or on disability pension were excluded from this study and were not among those lost to follow-up but outside the study scope.**

#“Our extensive nonresponse analyses have, nonetheless, shown that the data are broadly representative of the target population, although men, younger participants and those with more sickness absence were somewhat less likely to participate at baseline [21,62].” It would be important to further discuss how the limited participation of those on sickness absence might influence inference to the target population.

**RESPONSE: We have included more details on this. The differences were minor as reported in the published studies. However, it is likely that if we had all those in the target population as respondents, health would be slightly worse. Thus, the results could be somewhat conservative. To highlight, the cohort has been shown to represent the target population quite well, in several studies, so these differences are unlikely to distort the findings to any larger extent.**

#Generalizability to all employers should be discussed, as there might be differences between private and public employment in regard to access to occupational health services, health-related measures at the workplace, such as reimbursement and preventive measures, as well as supported employment for those with health problems, between the sectors.

**RESPONSE: As this cohort is from one employer, albeit the largest one in Finland, we cannot generalize the findings directly to private sector, or even to the entire public sector. However, it is difficult to see why the results would notably differ in other occupational cohorts in Finland if they were to examine developmental patterns in these outcomes among statutory retirees. Individual levels of physical activity or income are likely different, but as this is an epidemiological study, reporting findings at a group level, one could assume that broadly similar patterns might be repeated in public sector employees and potentially in private sector. For example, we have earlier extensively studied sleep, working conditions and sickness absence as well as social determinants of sleep, using both these HHS data before the participants retired, and also nationally representative large data (representative of all working-aged from different sectors, please see links to the studies here below). The results have been highly similar, suggesting that there are very similar associations between social and health-related determinants and outcomes, both in this cohort and using a nationally representative survey data. We have amended to revised Discussion to comment on these points (please see page 20 and 21). However, we prefer to retain these speculations minimal in the text, as we cannot study these current associations in a nationally representative cohort. These analyses can only be done with repeatedly collected data which are rarely available.**

**References:** [**https://bmcpublichealth.biomedcentral.com/articles/10.1186/1471-2458-12-565**](https://bmcpublichealth.biomedcentral.com/articles/10.1186/1471-2458-12-565) **&** [**https://academic.oup.com/sleep/article/37/9/1413/2416837?login=false**](https://academic.oup.com/sleep/article/37/9/1413/2416837?login=false)

#Generalizability outside the Finnish context should be discussed, as the social welfare system, access to healthcare, and public health measures differ in other countries/settings.

**RESPONSE: We have added discussion regarding potential generalizability outside Finland (please see page 21). Same points apply as above, as in general similar associations have been reported in different cohorts, using variable-oriented methods (studying associations between variables), as described in the Introduction and Discussion. One could assume that with repeated collected data among participants of the same age, there is no particular reason to assume why these developmental patterns and their associations with social and health-related factors would apply only in Finland. However, retirement age and retirement systems do notably differ, and this could make a difference. Also differences in health care systems are highly likely to play a role. We have added discussion to address the comment (please see page 21) but have kept speculations brief, as we cannot empirically prove them, and the manuscript is already lengthy.**

#Many of the covariates, such as obesity, alcohol use, and sleeping habits, can change over time. How did the author consider changes and possible interaction with the outcome measures?

**RESPONSE: It is true that the mentioned covariates can change. However, it would make the analysis extremely complex if we were to simultaneously study both changes in all our covariates (the time-variant ones) and changes in three outcomes. Additionally, the number of time points per participants should also be considered, as in the trajectory model. We agree this is an important point and have amended the Discussion to acknowledge this (please see page 22 and 23). It is quite common that determinants are taken before the outcome, and we trust it is the clearest procedure here. Thus, all the covariates are from Phase 1 and then developmental patterns in our three outcomes are examined from Phase 1 through 5. While some of the covariates may change, it is quite rare to lose weight or change from an unfavorable group to favorable or vice versa in any of the covariates. Therefore, it is likely that Phase 1 covariates help distinguish between participants with unfavorable and favorable circumstances (socioeconomic position and behavioral risk factors, for example).**

**Education is from young adulthood and was not asked again in any of the follow-up surveys. It emerged as a consistent determinant, and it is also closely linked to other time-variant covariates. Thus, having education partly helps control for example differences in obesity over time. Furthermore, we have shown in this cohort that differences in body mass index are clear from young adulthood, and remain highly similar from inclusion through follow-up (**[**https://karger.com/ofa/article/12/5/564/239657/Associations-between-Childhood-Disadvantage-and**](https://karger.com/ofa/article/12/5/564/239657/Associations-between-Childhood-Disadvantage-and)**), and that education is a clear determinant of higher body weight over time (**[**https://www.sciencedirect.com/science/article/pii/S2352827320303608?via%3Dihub**](https://www.sciencedirect.com/science/article/pii/S2352827320303608?via%3Dihub)**).**

**We have amended the revised Discussion to acknowledge these points about the role of time variant covariates (please see page 22 and 23).**

Conclusion
“This suggests that after retirement, one may not need excessive amounts of physical activity but a moderate, recommended level is likely sufficient to maintain good health.” The study design does not allow conclusions about causality, as the outcomes are measured at the same time. The authors have to be very careful of the interpretation of findings and not translate them into recommendations, given that the study design and method used are not adequate to study causal structures.

**RESPONSE: We fully agree that causal inferences are unwarranted and have revised the sentences to reflect the study design. We highlight though that the advantage of the design is that all outcomes are measures up to 5 times, and we aim to identify latent groups. This means that we would have been able to identify for example change in physical activity prior to change in health in some groups, should such a group have existed in the data.**

Reviewer #2:

1. Outcome of interest for selecting the trajectory models:
Based on the description of the methods, it is unclear to me which outcome was used to select the trajectory groups. As stated in Nagin et. al 2010 referring to both GBTM and GMM “Both methods are designed to identify cluster of individuals, called trajectory groups, who have followed a similar developmental trajectory on an outcome of interest”. Based on Figure 1, there are 3 outcomes of interest: General Health perceptions, Household income, and Leisure-time physical activity. It is unclear how the same 4 trajectory groups were determined for three different outcomes.

**RESPONSE: Thank you for your comments to improve our manuscript. We have clarified the Introduction and the Methods sections to be more explicit about the model (please see e.g., page 4 and 12). Thus, this is not a one outcome group-based trajectory model but a joint group-based trajectory model. The advantage is that it is possible to model development of different outcomes at the same time. We have for example earlier studied if anxiety, depressive symptoms and somatic symptoms jointly develop from adolescence to early adulthood (**[**https://bmcpsychiatry.biomedcentral.com/articles/10.1186/s12888-019-2203-7**](https://bmcpsychiatry.biomedcentral.com/articles/10.1186/s12888-019-2203-7)**). The model is explained in detail in this published paper, and we use it here in a similar way. In the Figure 1 of this current study, there are same people for each outcome for example for Group 1. Thus, it is possible to see how their physical activity, income and health jointly develops before and after statutory retirement (marked as 0 in the x-axis). As an example, individuals belonging to the Group 1 report good health, high levels of physical activity and first low, then sharply rising income that is stable after retirement. These trajectories are displayed in the Figure 1, alongside trajectories for Groups 2–4.**

**We prefer to retain the description in the paper brief and provide references for further details.**

2. Language- The paper would be easier to read and understand if the following issues were addressed:
Clarity: There are several very long sentences/ awkwardly worded sentences. Here is one example: “Following the joint development and co-occurrence of physical activity, health and income can help identify if there are groups of people who have little physical activity, poorer health, and low income, or if some groups are continuously better off, or if the development is distinct.”
Grammar: There are multiple grammatical errors throughout the paper. Here is one example: “which enables to approximate”, “The participants further provided an informed consent whether they agreed their survey responses”
I recommend rewording the phrase “somatic and mental diseases”.

**RESPONSE: As suggested, we have had our revised manuscript edited by a professional language Editor and a native speaker, who frequently revises English language of scientific articles in our field. We hope the text now reads better, including the sentences above. We also trust the grammatical issues are resolved during the language revision. This letter is not revised but we hope that the responses are understandable, and trust it is sufficient that the corresponding text in the manuscript has been revised by the professional language Editor.**

**Regarding somatic and mental diseases, we refer to the diseases the respondents listed diagnosed by a physician. Most of them are chronic diseases. Mental disorders are also requested and included.**

**We have reworded this and refer to physician-diagnosed chronic diseases and mental disorders. It is a bit lengthy but more accurate. Additionally, it is of note that they are self-reported as all survey items, so we cannot confirm that the participants actually have a specific medical diagnosis.**

3. Description of statutory retirement: While some background is provided in the Methods section, it would be helpful to have more information about statutory retirement in Finland, as many readers may be unfamiliar with this. Specifically, is retirement mandatory at a specific age or does it depend on the type of job? Additionally, to better understand the income of retirees, it would be helpful to have a description of the pension or retirement benefits that retirees in Finland receive.

**RESPONSE: We have clarified our description of the retirement system in Finland in the revised Methods section, as requested (please see page 6). The system is quite complicated, and it has also changed over time. You are correct to assume that there have been occupation-specific retirement ages and pension is dependent on the earlier salary. As this study has 5 time points, 3 outcomes and many social and health-related covariates, the paper is quite lengthy. While we fully appreciate that it is important to have more background, because systems differ between countries, we retained the descriptions short, to keep the text more readable and not to give too much emphasis on the retirement system. The main point is to focus on a cohort that has retired statutorily and study the development in their well-being before and after the transition. As the income is household income level, which more accurately describes the participants’ financial situation, their own pension can only be part of that income, as discussed in the manuscript. We also account for both age and retirement age in this study. Retirement age did not vary a lot, as we only focused on statutory retirees (disability retirement occurs earlier, but as it is by default health-related, and a rarer outcome, it would not be meaningful to add that here).**

**In this recent study conducted with the Finnish Centre for Pensions, we described the retirement system in a bit more detail:** [**https://jech.bmj.com/content/77/6/362.long**](https://jech.bmj.com/content/77/6/362.long)**. More details are also available online:** [**https://www.julkari.fi/bitstream/handle/10024/145447/statistical-yearbook-of-pensioners-in-finland-2021.pdf?sequence=1&isAllowed=y**](https://www.julkari.fi/bitstream/handle/10024/145447/statistical-yearbook-of-pensioners-in-finland-2021.pdf?sequence=1&isAllowed=y)

4. Leisure time physical activity:
a. For study reproducibility and generalizability, please provide the exact survey questions and response options for leisure time physical activity.
b. What was the timeline for the questions about physical activity? Were participants reporting on current activity or recalling previous levels of physical activity?
c. Which specific guidelines for physical activity were used to determine categories for levels of leisure-time physical activity. For example, 2.5 hours of brisk walking per week was categorized as “clearly below recommended leisure-time physical activity” in the discussion without citing a source. 150 minutes of physical activity per week is considered the recommended amount of physical activity for adults, according to some guidelines, including the World Health Organization.

**RESPONSE: Please find our responses to each of the sub-questions a, b, and c here below.**

a) **We have included our questions on physical activity and added more details on the measure, as requested. Full items are included in a new Supplement comprising all of the items of the study,**
b) **The activity is their current typical activity, asked to be reported during a typical week, over the period of previous 12 months.**
c) **We did not make any categories ourselves, as we used a joint group-based trajectory model to identify what type of development there is in the data in physical activity over the 22-year follow-up. The model identified these distinct trajectories, and from them, we checked the average MET-values in each group. These were compared to typical guidelines and recommendation for physical activity which have somewhat evolved since 2000, however, the basic message has remained, i.e. adults should do at least 150 min of moderate-intensity aerobic PA, or at least 75 of vigorous-intensity aerobic PA, or an equivalent combination of moderate-intensity or vigorous-intensity activity throughout the week for substantial health benefits (Borodulin and Anderssen 2023, Oja & Titze 2011, Fogelholm et al. 2005). This is clarified in the Discussion (please see page 26 and 27).**

**Borodulin K, Anderssen S. Physical activity: associations with health and summary of guidelines. Food Nutr Res. 2023;67. doi:10.29219/fnr.v67.9719**

**Oja P, Titze S. Physical activity recommendations for public health: development and policy context. EPMA J. 2011;2: 253–259. doi:10.1007/s13167-011-0090-1**

**Fogelholm, Mikael, et al. "Physical activity pie: a graphical presentation integrating recommendations for fitness and health." Journal of Physical Activity & Health 2.4 (2005).**

5. Sleep- Is there a reference for choosing 7 hours as the reference category? What happened to participants who slept between 7 and 8 hours? This level of the sleep category is missing from Table 1 and Table 2.

**RESPONSE: Thank you for this comment. Our sleep measure is but a covariate and it is rather crude. Unfortunately, sleep is asked to be reported only in full hours. This means that no category is missing but we initially chose those responding 7 hours as a reference group (average sleep), while those sleeping less (6 hours or 5 hours or less) as short sleepers and those sleeping more than 7 hours (8 hours, 9 hours, or 10 hours or more) as long sleepers. A vast majority slept 7 or 8 hours (72.4%). A problem with our categorization is that we do not know if 7.5 hours was reported as 7 hours or 8, or if 8.5 hours was reported as 8 or 9 hours. In sleep research, 7 and 8 hours are often combined, or even 6–8 hours, but as this cohort was employed at inclusion, it was extremely rare to report very short or long sleep. To have meaningful category sizes, and to somehow (with limitations), distinguish between short, average, and long sleepers, we initially chose this classification. If this was a sleep study, we fully agree we would need more detailed measures, or if we were interested in individual level sleep. However, for a covariate, and epidemiological purposes, we trust this variable works as a proxy for sleep quantity.**

**Following this and other comments, we have changed the categorization and now use 7–8 hours as a reference. It is in line with current recommendations for older adults (Hirshkowitz et al. 2015).**

**We have amended the revised Methods to be clearer in describing these measures, and all our covariates and outcomes (following other comments), please see page 9.**

**Hirshkowitz M, Whiton K, Albert SM, Alessi C, Bruni O, DonCarlos L, et al. National Sleep Foundation’s updated sleep duration recommendations: Final report. Sleep Heal. 2015;1: 233–243. doi:http://dx.doi.org/10.1016/j.sleh.2015.10.004**

6. Timing of measures
From the abstract and introduction, I understand that there were repeated surveys of participants at each of the 5 phases of the study. However, it is unclear in the methods section, if the “social and health-related covariates” were measured just at baseline, or at each of the 5 study phases. I understand the measurements that were taken at baseline (Table 1.), but were these measurements taken again at other phases of the study? I understand that some of these variables are unlikely to change, however, some variables such as amount of sleep, weight, and comorbidities may change during the study period. Additionally, the duration of time accounted for in the measurements is unclear. For example, what time period do the sleep and physical activity measures cover? Sleep the previous night, week, month, year? This is important in consideration of recall bias, and the stability of these measurements.

**RESPONSE: It is true that most of the items were repeated at each phase (1–5). This is important to reliably study developmental patterns in the outcomes. Some of the covariates such as education were not repeated as they are unlikely to change in this cohort. However, some of these covariates can change. It would make the analysis extremely complex if we were to study both changes in all our covariates (time-variant) and changes in three outcomes at the same time. Additionally, the number of time points per participants should also be considered, as in the trajectory model. We agree this is an important point and have amended the Discussion to acknowledge this (please see page 22 and 23). It is quite common that determinants are taken before the outcome, and we trust it is the clearest procedure for the purposes of this study. Thus, all the covariates are from Phase 1 and then developmental patterns in three outcomes are examined from Phase 1 through 5. While many of the covariates may change, it is quite rare to lose weight or change from an unfavorable group to favorable or vice versa in any of the covariates. Therefore, it is likely that Phase 1 covariates help distinguish between participants with unfavorable and favorable circumstances (socioeconomic position and behavioral risk factors, for example). Education from young adulthood emerged as a consistent determinant, and it is also closely linked to other time-variant covariates such as obesity. Thus, having education partly helps control for example differences in obesity over time.**

**Regarding time periods, we have clarified the revised Methods throughout and added a supplement of all the items, to explicitly state the time period for each covariate and outcome. For physical activity, it was previous 12 months, and respondents were asked to report their typical amount and intensity of activities during a typical week. For sleep duration, the question only asked how many hours (full hours) the respondents typically sleep in a day, without any time period specification. As we collected data 5 times, the response for outcome was always taken from the survey in question, thus recall bias is unlikely an issue. The respondents did not need to for example remember how they slept in childhood or when still working, or how much they used to exercise years back, but they reported their current activities and sleep.**

7. “All those who responded to at least two if the five follow-up questionnaires were included in this study”.
Given the following recommendation, please provide justification for the inclusion of participants with only two data points.
“In latent class modelling approaches for longitudinal data, at least three measurement time points are required for proper estimations, and four or five measurement time points are preferable in order to estimate more complex models involving trajectories following cubic or quadratic trends”. Trajectory Modelling Techniques Useful to Epidemiological Research: A Comparative Narrative Review of Approaches Clinical Epidemiology Nguena Nguefack, Hermine Lore; Pagé, M Gabrielle; Katz, Joel; Choinière, Manon; Vanasse, Alain; Dorais, Marc; Samb, Oumar Mallé; Lacasse, Anaïs
Vol. Volume 12, pp. 1205–1222, 2020.

**RESPONSE: It is true that 3 or more time points is often preferable to examine developmental patterns. In our study, only five participants (0.1%) had two time points, while a vast majority had all 5 time points (n=3178). Less than 10% had 3 time points and all the rest had 4 time points. We have now excluded those with only 2 points (n=5). As so few had but 2 time points, this decision made no effect on the identified and displayed trajectories. Please also note that most participants (87%) had at least 4 or 5 time points.**

**As an additional note, trajectory models are different in their specifications, for example how they use missing data or varying number of time points, depending on the software used, for example. Some are based on complete case data assumptions, while some can model trajectories with missing data and consider differences in individual follow-up times. With too strict criteria, we end up in a more selective sample, as loss to follow-up is typically somewhat selective. Even though we had a high response rate, we studied attrition for this revision (please see our new attrition analyses, Table S5). Another point is that when the numbers are lower, this has consequences on the model stability and category sizes. We have earlier published a paper comparing results from different models and software (**[**https://journals.plos.org/plosone/article?id=10.1371/journal.pone.0263810**](https://journals.plos.org/plosone/article?id=10.1371/journal.pone.0263810)**). The results between different software are similar but there are also differences as the models are not the same, although both identify trajectories. In all, we trust it is better to include a representative cohort.**

**We have reported the numbers of time points and average follow-up time (18.7 years) in the revised Methods (please see page 6), and overall improved the revised manuscript. Please note that differences in follow-up times do not affect these models.**

8. “Only a fourth had no physically strenuous work history” Please provide a percentage.

**RESPONSE: The exact percentage (22.4%) is reported in Table 1, alongside all the distribution for all our covariates. We aimed not to repeat the table contents in the text, as this a general requirement. Thus, we refer to the Table 1 in the text, and then summarize the results it displays. It would make the text quite heavy if were to add all the percentages for each covariate. We have now added this percentage, as requested, but otherwise we chose to highlight a few key ones only.**

9. “Thus, people with obesity had a 23% higher likelihood to belong to the Group 1, as compared to people with normal weight.”- Is this based on weight at baseline or is this time-varying?

**RESPONSE: This estimate (23%) is from the multinomial logistic regression model, and all the estimates reported in the Table 3 are average marginal effects (AMEs). We also report in our Supplementary Table 3 all the odds ratios (ORs) from the same model. The reason to report AMEs in place of ORs is that for AMEs, we do not need to choose one category of our outcome as reference, but we can study how social and health-related factors are associated with each category of the outcome. ORs are always compared to the chosen reference and depend on it and the sizes of the groups, for example. Thus, there are no estimates for the reference group. The ORs can exaggerate the associations and they are also more difficult to interpret. AMEs instead are concrete, as they are interpreted as percentage points differences. Thus, people with obesity (at Phase 1) have a 23 percentage points higher predicted probability to belong to the Group 1, as compared to people with normal weight. All the covariates are from Phase 1, thus preceding the outcome (trajectories from Phase 1–5).**

**We have clarified and corrected how we refer to the differences, as they are percentage points, not percents. Further details are given in the text, references to the model and how AME compare to OR. Please also see our response in the letter regarding choosing the Phase 1 covariates.**

10. “While high levels of physical activity are consistently linked with better health, this study also suggests that recommended level of physical activity is likely sufficient to maintain health in aging and older adults.” Please address the potential for a bidirectional relationship between physical activity and health, for example, those with more physical activity may report better health, however, those with better health may be able to be more physically active.

**RESPONSE: Thank you for this comment. We have improved our manuscript to clarify the results. We appreciate that bidirectional associations are possible in general. Thus, at any time point, health can affect the amount of physical activity, and physical activity to perceived health. In this design, we, however, ‘let the data talk’ and identified latent developmental groups. This means that over the 22-year follow-up (max), there are these groups of people, who have distinct development in their well-being, as indicated by leisure-time physical activity, health, and income. As some of the respondents consistently reported high levels of physical activity and better health, we could identify this latent class in the model. We could identify a group of participants who had at Phase 1 good health and high levels of physical activity, and this pattern continued over time. If there were other types of response patterns and associations, the classes would look different. Thus, the model is not based on any categorization nor a direction of the association determined by us, but the results reflect actual latent groups in the data. It is acknowledged that they are approximations of the true development, thus some participants can be misclassified.**

**In sum, this means that we are not suggesting any direction of associations, but the results illustrate how these outcomes jointly develop over the follow-up.**

**We reported average posterior probabilities in our Supplementary material showing that misclassification is likely a minor issue.**

**This response is to clarify the key differences that person-oriented methods have as compared to variable oriented methods (studying e.g., bidirectional association between variables and time points). In other words, as the model is designed to identify joint developmental patterns (latent groups) in the chosen outcomes, the model should identify the links in time between physical activity and health, if they developed together. Same applies to income, as we studied the joint development of all these outcomes.**

11. “Among the potential factors, education and obesity were key factors explaining the patterns.”
Education likely did not vary over time during the study, given that the participants were retirement age, however, obesity, specifically weight is a time-varying covariate. Were changes in weight over time accounted for? Also, there is a potential for a bidirectional relationship here, “Reporting three or more somatic and mental diseases was associated with a higher likelihood to belong to the Group 1 (26%)”. A higher number of somatic and mental diseases may be associated with an increased likelihood of obesity.

**RESPONSE: Education was only asked at Phase 1, and it is true that it is mostly time-invariant in this cohort of older and retiring employees. Obesity can change (people mainly gain weight), but we have examined obesity trajectories in this cohort in our previous studies. The results show that the baseline obesity is the most notable determinant of future development. Thus, the are clear differences at baseline, but they remain stable, and the differences observed at baseline remain. Thus, including Phase 1 measure is likely to be able to distinguish between participants living with and without obesity. Please see Figures 1 & 2 of this study https://www.ncbi.nlm.nih.gov/pmc/articles/PMC6876612/ for reference:** [**https://www.ncbi.nlm.nih.gov/pmc/articles/PMC6876612/figure/F1/**](https://www.ncbi.nlm.nih.gov/pmc/articles/PMC6876612/figure/F1/)**, women &** [**https://www.ncbi.nlm.nih.gov/pmc/articles/PMC6876612/figure/F2/**](https://www.ncbi.nlm.nih.gov/pmc/articles/PMC6876612/figure/F2/)**, men). It is also of importance that education is a key determinant of the development of obesity as shown also in this cohort:** [**https://www.sciencedirect.com/science/article/pii/S2352827320303608?via%3Dihub**](https://www.sciencedirect.com/science/article/pii/S2352827320303608?via%3Dihub)**. Thus, controlling for education partly addresses the change in obesity. We acknowledge though that for those whose weight notably increases, there could be consequences for physical activity and health. Education is also strongly linked to health, which further highlights the significance of adjusting for education, helping to consider the diseases.**

**Please also note that regarding chronic physician-diagnosed diseases, the most important point is to consider the baseline situation, when our health outcome is first measured. Thus, when our outcome is change in health, it is important to consider then inclusion time, but we cannot simultaneously have two overlapping measures of health. We have made clarifications in the Methods for our measurements, and we hope that the section is no clearer and the choices justified.**

12. “While high levels of physical activity are consistently linked with better health, this study also suggests that recommended level of physical activity is likely sufficient to maintain health in aging and older adults.” Please provide more details about what findings from this study this statement is based on.

**RESPONSE: We have clarified this sentence, as requested. The sentence could be misinterpreted, and it is not intended for causal inferences. We highlight though that the advantage of this design is that all outcomes are measures up to 5 times, and we aim to identify latent groups. This means that we were able to identify joint developmental patterns of physical activity and health (and income). The trajectories display that there are groups of people who report high levels of physical activity and health but also a group of people with less high level of physical activity (yet within recommendations), who also report good health. The idea is that the model identified latent groups in the data, and we did not make any categorization or decisions before the joint group-based trajectory modeling.**

13. “Physical activity typically likely declines with aging and is important for health [11] but changes in income did not develop jointly with changes in health in all groups.” This sentence is not very clear, and it would be helpful to separate out the findings for physical aging and income.

**RESPONSE: We have revised the sentence and hope that it now reads better. A native English speaker, who is a professional language editor of scientific papers has also edited the entire manuscript for proficiency in English.**

14. “We also identified large groups of people – two thirds of the current population - with recommended and high levels of physical activity, continuing to have good health.” Again, please address here the potential for a bidirectional relationship between physical activity and health.

**RESPONSE: The sentence is based on the results of our joint GBTM model, which identified four distinct group with the largest groups reporting good health with recommended levels of activity. The model let the data talk, and it is not about causality or bidirectional associations. Thus, the results above are not suggesting any particular direction of the association, but joint developmental patterns. To correctly interpret the results, we hope our revised manuscript and these responses give more details about the model. We particularly hope that the clarification regarding the model and its assumptions and specifications help interpret the results. Thus, we can show how these outcomes develop jointly over time, and the groups are latent groups, identified by the model. It is of note that they are self-reported data which means that we cannot rule out any reporting tendency, which is common to all survey-based data. However, it would be quite difficult to collect large data for over two decades, with any other method. The results must be cautiously interpreted, and causality is not assessed. The trajectories are always an approximation of true development. We have amended the Discussion to consider bidirectional associations, clarifying that they are in general possible, and we cannot rule out reporting bias. The joint-GBTA is, however, different from variable-oriented longitudinal analyses and this affects also how this question can be addressed.**

Reviewer #3:

Thank you for the opportunity to review the manuscript titled: “Joint trajectories of physical activity, health, and income before and after statutory retirement: a 22-year follow-up”. The study covers an interesting and relevant topic and employs a sophisticated analysis technique. The authors should be lauded for having collected a large volume of data spanning up to 22 years. Nevertheless, I am concerned about some of the analytical and methodological choices, particularly considering this study was not pre-registered. Please find below several suggestions and comments, which I hope will aid you in improving this manuscript:

**RESPONSE: Thank you for your comments and suggestions to improve our study. Please find here below our point-by-point response to each of the comment raised. We have especially paid attention on the more detailed description of the methods as well as conducted some additional analyses e.g., on attrition. We have also added a new Supplement Table S5 including the results from those analyses.**

**Please also note that while we fully appreciate your comment regarding pre-registration, it is extremely rare for these types of observational studies. We have collected these data for over 22 years, as you kindly noted, and published hundreds of papers using these data. None of the published original observational studies (similar to this one) was pre-registered. It is not feasible to publish a pre-registration for each study, nor a common practice for any observational cohort, where it is possible to study a vast number of different hypotheses or research questions with hundreds of different variables collected at each phase. However, for example for systematic reviews or trials or studies that do require such pre-registration, it is a different question. For observational study such as this, we aim to report the methods as accurately as possible and have made our best effort to clarify the methods in the revised manuscript, following all the advice and comments from you, all the Reviewers and the Editor.**

General comments:
The English language should be improved to ensure that an international audience can clearly understand your text. An example where the language could be improved is (introduction): “However, the above previous variable-oriented studies have not considered latent groups or examined how indicators of well-being develop together, including changes over a major life transition, statutory retirement, following-up same people from their mid- or later careers until older age”. The current phrasing makes comprehension difficult. I suggest you have a colleague who is proficient in English and familiar with the subject matter review your manuscript, or contact a professional editing service.

**RESPONSE: As suggested, we have had our revised manuscript edited by a professional language Editor and a native speaker, who frequently revises English language of scientific articles in our field. We hope the text now reads better and can be understood, including the sentence above.**

Could you comment on whether it is problematic that the data collection for phase one spanned three years (instead of one year for subsequent phases), meaning that the time difference between phase 1 and 2 ranges from five to seven years?

**RESPONSE: We appreciate the comment and have clarified the Methods section regarding this comment. We do not see any issue here. The data collection for the 1^st^ Phase spanned three years because we wanted to have sufficiently large cohort to follow-up. We included all employees of the City of Helsinki, who turned 40, 45, 50, 55 or 60 years in either 2000, 2001 or 2002. This was because at those ages, the employees were invited to a health-checkup (5-year age groups). If we only had year 2000 as baseline, we would have only had about 3000 participants in our cohort. Over time, in each cohort, there is attrition and then it would be problematic to for example do any subgroup analyses or have multiple outcomes and determinants. The Phase 1 participants were homogeneous as they all fulfilled the same inclusion criteria regarding their age and being employees of the City of Helsinki. We also have health-checkup data for them, but not for all as the check-up was not mandatory. For the purposes of this study, we only used survey data for maximum numbers and because health-check up data are only available at inclusion year, and it is not complete. After Phase 1, we have sent the surveys to all who responded at Phase 1 and kept the follow-up the same for all for clarity.**

It would be beneficial for the reader to know how you defined retirement. Perhaps also mention whether there is a mandatory retirement age in Finland.

**RESPONSE: We have clarified the Methods section regarding this comment. We agree it is important to clearly state this, as retirement systems vary hugely between countries (please see page 5 for retirement definition and page 6 for system description). In brief, we only focused on statutory retirement. The retirement age varies and earlier, it may also depend on occupation. The retirement system changed early on during the follow-up and now it is possible to retire flexibly, after the age of 63. When turning 68, it is in general mandatory to retire, however, it is possible to continue some work. The system is quite complex, so it is difficult to explain it without making the manuscript overly lengthy, confusing the readers. We have thus provided some references for further details for those who are interested in the Finnish system. Our definition is based on the respondents’ own report about when they retired and whether it was due to old age (statutory retirement) or disability retirement or other reason. Most retire statutorily and as disability retirement is by default preceding an illness, it was excluded here. Thus, health trajectories of people retiring due to disability greatly differ and also, they retire younger. Their numbers are small and thus we could not study them separately here. It would also double the amount of all tables and figures and necessitate another type of Introduction and Discussion. Therefore, we feel it is better to study disability pension separately, should the numbers suffice. In addition to added descriptions in the revised manuscript, we also added our survey items showing how the retirement transition was asked from the participants.**

I am concerned about your decision to include all participants who completed two surveys. This means that some participants are followed for 5 years and others for 22 years. Greater changes are likely to occur if participants are followed for a longer period of time and older participants are more likely to drop out due to death or other factors during that time. Could you perhaps explain how you addressed this difference? Further, and this is a minor point, I would argue that Phase 1 is the baseline survey, which means that the four subsequent surveys, or phases, are the actual follow-ups, meaning there are only four follow up phases.

**RESPONSE: We appreciate that referring to completion of two surveys only could arise concern, and we are sorry we missed explaining this better initially. Thus, in our study, only five participants (0.1%) had but two time points, while a vast majority had all 5 time points (n=3178). We have now excluded also those with time points only. As so few had but 2 time points, this decision made no effect on the identified and displayed trajectories. Less than 10% had 3 time points and all the rest had 4 time points. Most participants (87%) had at least 4 or 5 time points. If we only included those with all time points, this would create other problems. Most importantly, the data would be more selected, as those who are healthier and more advantaged, are more likely to participate at each follow-up, while those with health problems, lower socioeconomic position and less activity are typically overrepresented in attrition in surveys such as this one. This means that with strict criteria for inclusion, our trajectories would represent the better off. We trust it is better to use as much data as possible to avoid selection and have a more representative cohort. We have, however, improved our Discussion to better consider this choice. We have also added figures regarding the average follow-up and the numbers of people with 3–5 time points. As a strength, the participants have responded well to follow-up surveys, and thus attrition is not a major issue, and for the majority, we do have several time points. We have also added a new supplementary table 5 on attrition for further confirmation.**

Do you have a code/number available for the ethics approval? I think it would be helpful for this to be mentioned here.

**RESPONSE: At the study start (2000), the ethical committee did not provide any code, just the approval. For this type of study, it was not, and it still is not required to have any ethical approval as we do not do any tests on individuals. They only fill in the survey. The ethical approval is for the study plan in general, from the Faculty of Medicine. The City of Helsinki has given as permission to collect the surveys among their employees.**

I think the outcomes section could be greatly improved by providing more detail. Ideally, provide the exact wording of the assessment, either in the text or an appendix, so the reader knows exactly what you assessed and over what period.

**RESPONSE: We have improved the outcome section to more detailed regarding our outcomes. We hope the details are helpful and provide the exact information, as requested. We now provide an attachment with all the items used in this study, translated into English. Please note that the questionnaire was mailed in Swedish or Finnish and thus English versions are either translated by us or from original surveys for validated international measures such as RAND-36, where we took the health measure (perceived general health).**

It seems odd you would assess leisure time physical activity by solely asking about walking, brisk walking, jogging and running or equivalents. Leisure time physical activity is a broad category and may comprise gardening as well as heavy weightlifting, for which it may be difficult to find these equivalents. Overall, this would not be an adequate representation of leisure time physical activity.

**RESPONSE: It is true it may be difficult for the participants to find all suitable equivalents for their different activities. The question provided a quite long introduction and guidelines to assist them. We have improved the description in the Methods to provide the readers with this same information. We also acknowledge in the revised Discussion that we may still miss some lower intensity activities, due to difficulties in finding all the suitable equivalents. If this study was focused specifically on physical activity, we would have preferred a more detailed list of survey items on different activities as well as accelerometer measurements. However, this is a broad health survey conducted since year 2000, where we focus on socioeconomic inequalities in health, working conditions, health behaviors and different health outcomes. The survey was a lengthy one and thus it is also a compromise what to include, to have valid measures but not too lengthy survey. The chosen physical activity measure has been used in well-established Finnish Twin Cohort study (Kujala et al. 1998, Waller et al. 2008) as well as other Finnish cohort studies (Stenholm et al. 2016).**

**Gardening and other yard work are quite common among midlife and older adults in general in Finland, however, likely quite rare in this cohort, as it is rare to have gardens in Helsinki (people live mainly in apartment buildings). Weightlifting or strength training in general are also popular Finland, however, these participants are of an age cohort where weightlifting is not a common hobby. They are likeliest to walk, ride a bike or ski in the winter, or some may do some other aerobic activities (Wennman et al. 2021, Wennman & Borodulin, 2019). As they are older adults (the oldest ones are 82 years at 2022), walking is most common type of activity. Additionally, as we do study individuals but groups, it does not need to distort the findings, if we miss some type of activity. The items are likely to provide hierarchy, and identify people with low, intermediate, or high activity (which in reality can be even higher). Furthermore, our physical activity measure has shown to predict mortality and different health outcomes (Lahti et al. 2013, Lahti et al. 2014). Finally, while no measure of physical activity is superior, we hope to have better acknowledged the limitations of this one in our revised Discussion (please see page 21 and 22).**

I think the “Health” section would be more appropriately labelled as self-perceived health. I think you cannot use this synonymously with health. This has implications for the remainder of the manuscript, especially the discussion section.

**RESPONSE: We fully appreciate this point, and aimed to state this clearly when our health measure was first introduced. However, to save words and for clarity, we used the name of the survey measure. Please also note that all our measures are self-reported, and we think it would make the paper quite tough to read, if we repeated self-reported each time we mention any of our measures. If we only repeated it for heath, it might also strike a bit odd. More importantly, it is not unfortunately possible to use self-rated (or self-perceived) health as it is a commonly used term for a single item from the RAND-36 (SF-36) health questionnaire, used in numerous studies. Our health measure is a dimension of the same survey inventory with multiple items. We need to use the term that is recommended by the developers of the measure, and used in the literature for the subscale, for comparability and consistency. We have, however, clarified this in the revised manuscript and aim to state clearly that all the items are self-reported in the surveys (except for occupational class, which is in part from the personnel register, as explained). As we use survey data, we hope it is also implicitly clear that the measures are self-reported. This is now also explicitly stated in the revised manuscript.**

Please, could you provide more information on the education categories and how these were derived?

**RESPONSE: More details regarding the question about education are available in the new supplement listing all the items used in this study, including education and all the response categories. It was very brief in the survey but is has been used in 100+ studies and it can distinguish between people with low, intermediate, or high education. This is important, as educational system has completely changed and thus details of the older system are not familiar to current generation, and they are not comparable to current system. However, the hierarchy still works, and the point here was to be able to distinguish between the level of education, not the actual education itself (such as how the system was).**

You state: “We also adjusted for physically strenuous work before retirement transition and age during the first study wave.” How was this done? How did you define physically strenuous work?

**RESPONSE: We have added more details and a supplement of the survey items to better describe this covariate. It is a single item from the survey which has worked well in distinguishing between participants who have had physically heavy or light work or no physically strenuous work. In the City of Helsinki, there are still many jobs where people do physically heavy work, such as in health and social care sectors and to some extent in education (day care of younger children). Our interest is whether physically strenuous work is linked to distinct development in our outcomes. Changing jobs is rare as people’s main route out of paid employment is for statutory retirement.**

Why was a cut-off of 7 hours per day chosen to assess sleep?

**RESPONSE: Sleep duration has been studied extensively, and in general short and long sleep are associated with adverse health outcomes while for older adults, 7–8 hours is recommended. It is true that this is heterogeneous, as for a minority, even 5 hours maybe sufficient, while other may need 9 hours. However, most sleep around 7 or 8 hours (72.4%) and our item did not allow for more exact analyses of sleep duration but full hours. For meaningful group sizes for analyses (epidemiology), we initially chose a common cut off that also worked in this data. As this is not a sleep study (where more detailed sleep measures would be needed), and we are not focusing on the developmental patterns in sleep, we trust that as a covariate, this variable could adequately classify participants as short, average, and long sleepers. We have now made a new categorization following also current sleep recommendations for older adults (**[**https://www.sciencedirect.com/science/article/abs/pii/S2352721815000157**](https://www.sciencedirect.com/science/article/abs/pii/S2352721815000157) **& http://dx.doi.org/10.1016/j.sleh.2015.10.004). The reference category is thus now 7–8 hours of sleep. Please see page 9 for changes in the Methods regarding sleep duration.**

Is the BMI measurement adjusted to include participants who can be categorised as underweight?

**RESPONSE: In this cohort, there were less than 1% of participants with underweight. As BMI was only a covariate, we did not want to redundantly lose participants or delete people with underweight. Therefore, we combined them in the category of normal weight. The group was too small to study it separately and merging it does not change the results due to the very low number of people with underweight. We have revised the Methods to include description on the BMI measurement and the procedure with BMI<18.5 (please see page 9).**

Could you explain why never or ex-smokers were conflated into one category?

**RESPONSE: We agree that often never and ex-smokers are studied separately. However, as smoking was a covariate and to avoid too many categories with low numbers, we initially chose to focus on current smokers only. This means that the reference group included ex-smokers. This has been done in many studies where smoking is only a covariate and adjusted for the purposes of controlling mainly the effects of current smoking. It is possible that the heterogeneous reference category may to some extend then attenuate the contribution of smoking, because ex-smoking still can contribute to the development of the outcomes.**

**We thus created a new variable with three categories: current smoking, ex-smoking and never smoking.**

**We have revised Methods and all our tables accordingly (please see page 9 and each table).**

Could you elaborate why you chose binge drinking behaviour instead of alcohol consumption at various levels? There appear to be few participants actually engaging in binge drinking behaviour, which makes me contemplate the usefulness of this.

**RESPONSE: We appreciate this point and have added justification for this in the revised paper. We have extensively studied drinking in this cohort and as the cohort, and the public sector, is female-dominated and as the participants were employees at baseline, heavy drinking was rare. It is likely that those with most severe alcohol problems were not working (unemployed or on disability pension), or they did not respond to the survey, or they underreported their drinking. In our previous study, we pooled data from 129,942 adults across 12 cohort studies (1.73 million person-years at risk) and showed that binge drinking is a key factor regarding losing healthy life years (Nyberg et al., 2022). While heavy drinking also reduced the number of disease-free life years, those with both heavy and binge drinking had the shortest disease-free life span. Binge drinking was chosen as a covariate as it is a clear and undisputable risk factor, it is never recommended, and it is against for example Current Care Guidelines. Again, as it is a covariate only, we think it may best capture the potential harmful contributions of drinking to the development of our outcomes. As we have a large number of both social and health-related covariates, we further had to more selectively choose the ones to use.**

**We have clarified the choice of this measure in the revised Methods and provided reference to the previous study (please see page 9).**

**Reference: Nyberg ST, Batty GD, Pentti J, Madsen IEH, Alfredsson L, Bjorner JB, et al. Association of alcohol use with years lived without major chronic diseases: A multicohort study from the IPD-Work consortium and UK Biobank. Lancet Reg Heal Eur. 2022;19: 100417. doi:10.1016/j.lanepe.2022.100417.**

Regarding the disease count, what was the time span that was assessed? Was it the five years prior?

**RESPONSE: All diseases were asked as ‘Has a doctor ever diagnosed you with the following diseases’ and then a list of diseases. Many of them are chronic such as diabetes. There is no time span, but the list of responses reflects what the participant listed as a doctor-diagnosed, typically a chronic condition, at the study inclusion. We have revised the description of this variable and have renamed it following another comment/suggestion (please see page 9 and each table).**

Overall, the outcomes section could greatly benefit from more detail pertaining to how details were assessed and over what time span.

**RESPONSE: We have clarified the outcome section to provide more details about what was assessed (please see page 7 and 8). The time span and more details, where asked, are available to be checked from the new Supplement listing all the survey items used in this study.**

Were there any missing data and if so, how were these handled?

**RESPONSE: Missing data were not typically common (below 2% per item mostly). There are always missing data in surveys, as the respondent can choose which items to fill in and which ones they choose to leave unanswered/ empty, by choice or by mistake (miss them). We have described in the revised Methods, how we handled these missing data. As we have a large number of covariates, we needed to handle the item missing, as otherwise the data might be selected, and the statistical power could suffer. This could even distort the conclusions. Typically, different items are missed by different people, which means that per item, missing is not an issue but when one has 20 variables, item missing accumulates and then the total number can be higher, resulting in selected data if nothing was done.**

**For outcomes, missing % was low and respondents were excluded from the analyses, if they had missing data on the outcomes. For covariates, there was no missing data for gender or age, and very few for marital status or education. For BMI and sleep duration, a few percent had missing data these were replaced by the mean BMI and mean sleep duration. For other covariates, the small numbers of missing values were included in the reference group, following previous procedures with these data (we have hundreds of studies publishes since 2000 with these data, most of them listed on our website). Reference group is used as then the effect of the imputation is conservative, thus we do not exaggerate any associations. As the numbers missing were in general very low (below 2% per item), this decision is unlikely to distort the results.**

**We have amended the description of the missing data throughout the revised Methods section.**

It is not clear to me how you determined the change from employment to retirement. Presumably this varied between individuals. Did you follow individuals as they aged, i.e., as their age increased irrespective of phase, or did you use subsequent survey participations as an indicator of time passed. The latter may be problematic, given the range of ages represented. This question may be rooted in a misunderstanding of the analytical methods employed, but nevertheless, it is worth clarifying this issue for the reader.

**RESPONSE: We have clarified in the manuscript, how retirement was determined (please see page 5 an 6). In brief, all participants were employees of the City of Helsinki at inclusion. We started a cohort of midlife and aging employes, as we wanted to study them before and after the retirement transition, a major life event. The follow-up surveys were mailed to all Phase 1 respondents, irrespective of their current employment status (still working or retired). We then asked in each survey their employment status. We used the responses in the surveys to determine when they had retired, and then all participants were selected in this study on the basis of them reporting to have statutorily retired. At Phase 5, only about 10% were still working, and they were not included, but for all others, we could have a retirement year from that survey.**

**The follow-up time before and after retirement varies between participants, but the model is not affected by this. We wanted to present the trajectories so that they clearly display the retirement transition and the outcomes before and after that. The other opportunity would be to use for example age or Phase as x-axis, but then we would not know when the retirement occurred. Also we consider age in the models and focus of this study is not what happened at each Phase but what happens before and after retirement transition.**

Table 1 indicates everyone took part in Phase 1, which is seems at odds with your stipulation of participants needing to have participated in two follow up surveys, which may exclude phase 1. Please can you clarify this? Were any participants added at subsequent phases?

**RESPONSE: All participated at Phase 1, as otherwise they would not be part of this cohort. They were then working at the City of Helsinki. Follow-up surveys were only mailed to those who participated at Phase 1. Thus, we did not add any new participants later on, but this is a follow-up of the same individuals throughout the study period. However, for the purposes of this study, we only included those who retired after Phase 1. During the follow-up of 22 years, about 90% of the initial respondents retired. We have amended the description of the data and methods for this revised version of our manuscript.**

Please include Figure 1.

**RESPONSE: The Figure 1 is included in the manuscript. It was asked to be downloaded as a separate file in the submission system and hence it appears in the end of the pdf file. In the manuscript, it is requested to add its title only.**

Can you comment on why household income is not represented in tables 1-2?

**RESPONSE: The Table 1 displays all our social and health-related covariates. The figure 1 displays the outcomes: physical activity, health and household income. It would be complicated to add all our outcomes in the Table as they are measured 5 times and person-oriented model (trajectory analyses) is used to identify latent groups. Thus, if we present for example distributions of all our 3 outcomes at 5 phases, the table will be very long. More importantly, it then would not reflect our aim nor the model done, as the trajectories are displayed around retirement transition, not by phase. Moreover, the key idea of using trajectory analysis is to identify latent groups and distinct developmental patterns in these outcomes (we let the data talk, and not classify the outcome ourselves). Covariates are classified by us and used in the cross-tabulation (Table 2) and in the multinomial logistic regression (Table 3 and Appendix Table), which is back to the variable-oriented approach, where we use as outcome, the latent groups identified in the trajectory model. Thus, our Figure 1 is providing the descriptives for the outcome, and Table 1 for covariates, and then in Table 2 and 3, we study the associations between covariates and trajectory group membership. Distributions of the outcome variables (and other variables) are now available in the new Supplement Table S5 about attrition.**

You discuss increases and decreases in various measures. Could you comment on the size and magnitude of these?

**RESPONSE: We have aimed to improve the results to comment on these. The Table 3 and the Appendices are quite large, and it would make the Results quite heavy if we repeated a lot of estimates. We have tried to pinpoint few most important ones, to avoid repeating the tables too much. We hope a few examples are helpful, and then for those interested, more details are available in the tables. The advantage of the AMEs is that they are very concrete, as they show the probability to belong to a group as percentage points differences. We also included odds ratios, but they are dependent on the outcome and size of the group, for example.**

What was the average number of years that participants were followed up for?

**RESPONSE: We have added the average follow-up time, 18.7 years, and more details on the data collection, as requested in other comments as well (please see responses above). In all 79.4% had a 20-year follow-up, 15.9% had a 15-year follow-up, and 4.7% had a minimum of 10-year follow-up (3 time points), please see page 6.**
