# Supplementary material for: Joint trajectories of physical activity, health, and income before and after statutory retirement: A 22-year follow-up
Source: PLoS One. 2025 Jan 29;20(1):e0317010. doi: 10.1371/journal.pone.0317010 (PMC11778762; doi:10.1371/journal.pone.0317010)
Supplement: Attachment — Submitted filename: Response to Reviewers_revision_2.docx [file pone.0317010.s003.docx]

**Reviewer #3**

Thank you for the opportunity to re-review this interesting manuscript on the joint trajectories of physical activity, health, and income before and after statutory retirement.

The English language has been widely improved throughout the manuscript, as has the manuscript overall. Nevertheless, some issues remain that need to be addressed.

I think one of the main issues with this study is that the variables you set out to examine in the introduction do not adequately map on to the actual variables in your data set without limitations. Given the nature of the data I can follow the authors’ arguments for operationalising the variables and data in the way that they did. Nevertheless, these compromises may merely mask some of the underlying more fundamental issues with some of the variables. In the case of leisure time physical activity, for example, the authors acknowledge that they may miss certain activities due to the way this was assessed, but suggest that due to this being a co-variate, it does not invite the same level of scrutiny. I would argue that being a covariate does not mean that a different level of scrutiny should be applied, especially given the fact that PA does form part of the groups that were identified and is mentioned in the title as a main factor. This also applies to BMI and especially the sleep assessment. While the authors do acknowledge the limitations for PA assessment in the discussion, this should be made similarly clear for BMI and sleep assessment.

**Response**: We agree that self-reported questionnaire data always have certain limitations and measured data could provide more detailed information. However, it is quite difficult to obtain measured data for thousands of participants across long time periods. PA is one of our outcomes and of more importance than covariates, as we model its development over time. Therefore, we have aimed to acknowledge the limitations of the measures. Yet no measure is superior (van Poppel et al. 2010). BMI and sleep were used only in the multinomial model to study their associations with trajectory membership, however, we agree it is important to be open about their limitations for correct conclusions regarding their importance to the developmental patterns. For instance, our Phase 1 survey data were compared to measured health check-up data and self-reported and measured BMI showed relatively high correlation and similar associations with subsequent sickness absence (Korpela et al. 2013). Previous research has shown that self-reported sleep duration is a crude measure and people tend to over-estimate their sleep duration showing moderate correlation with actigraphy measures for instance (Lauderdale et al. 2008). In turn, people with insomnia may under-estimate their sleep (Sivertsen et al., 2009). Yet self-reported sleep duration likely provides reasonable estimates for distinguishing different groups i.e. short, average, and long sleepers, and, for instance, sleep duration has shown association with sickness absence using nationally representative data (Lallukka et al. 2014). We have extensively discussed the limitations regarding our self-reported physical activity measure, and improved discussion on our covariates i.e. BMI and sleep as requested.

*References:*

Korpela K, Roos E, Lallukka T, Rahkonen O, Lahelma E, Laaksonen M. Different measures of body weight as predictors of sickness absence. Scand J Public Health. 2013 Feb;41(1):25-31.

Lauderdale DS, Knutson KL, Yan LL, Liu K, Rathouz PJ. Self-reported and measured sleep duration: how similar are they? Epidemiology. 2008 Nov;19(6):838-45.

Lallukka T, Kaikkonen R, Härkänen T, Kronholm E, Partonen T, Rahkonen O, Koskinen S. Sleep and sickness absence: a nationally representative register-based follow-up study. Sleep. 2014 Sep 1;37(9):1413-25.

van Poppel MN, Chinapaw MJ, Mokkink LB, van Mechelen W, Terwee CB. Physical activity questionnaires for adults: a systematic review of measurement properties. Sport Med. 2010;40: 565–600.

Sivertsen B, Øverland S, Pallesen S, et al. Insomnia and long sleep duration are risk factors for later work disability. The Hordaland Health Study. J Sleep Res 2009;18:122-8.

On a related note, not referring to self-reported health as such is misleading. This is especially true for health as there are vast differences between subjective and objective reports. I think this especially applies to your discussion, where you use the term “health”, without qualifying this to be self-reported. I understand that all of the measures in this study are technically self-reported. Therefore, perhaps a better term would be self-rated health or similar (to distinguish from other variables) that encapsulates the fact that participants were asked to estimate their own health and disease susceptibility, rather than listing conditions or using a composite measure to indicate health.

**Response**: We have now used general health perceptions throughout the manuscript to avoid confusion. The RAND-36 has 8 dimensions that assess different aspects of physical and mental health: physical functioning, role limitations due to physical health problems, role limitations due to personal or emotional problems, emotional well-being, social functioning, energy/fatigue, and general health perceptions (Hays 1993 & Hays 2001). The term suggested by the developers of the RAND-36 is preferred to self-rated health, as self-rated health is commonly used to refer to a single item measure derived from the same RAND-36 (the first item of the measure), and referenced in the paper (references 31, 32 and 33). This could be confusing and make comparisons between studies difficult. We trust that adding the full-term general health perceptions clarifies the measure is self-reported, as it refers to perceptions. Moreover, the Methods are specific and explicit about the nature of the data and that all items are from surveys. We also explicitly state in the revised Methods section that the subdimension is self-reported. Using the term as measured by general health perceptions makes the text longer and a bit harder to read, as the term cannot be as conveniently used together with good or poor, as before (good health vs. poor health), however, we hope the term is now clear and less misleading.

*References:*

Hays RD, Sherbourne CD, Mazel RM. The rand 36-item health survey 1.0. Health Econ. 1993;2(3):217-27.

Hays RD, Morales LS. The RAND-36 measure of health-related quality of life. Ann Med. 2001;33(5):350-7.

The self-report nature of retirement is another significant issue and the implications associated with this should be made clearer in the limitations section of the manuscript. I appreciate the difficulty in defining retirement, especially considering changes in legislation, but especially given the complexity of the system there is great potential for confusion, even among participants themselves. It is, for example, not clear whether participants would consider themselves retired when in they were “semi-retired”, perhaps still working, but only part time and in a different job. These limitations need to be discussed and the implications evaluated.

**Response**: We have now clarified that only full-time statutory retirees were included. In Finland, full-time old-age retirement is probably self-reported with high accuracy due to the structured pension systems, and clear retirement age thresholds. However, we acknowledge that some uncertainty exists in situations such as where people are retired due to old-age and still continue to work part-time. As they still continue to have work-related exposures and may for example commute to work, they are not directly comparable to full time retired participants.

Please refer directly to the supplementary materials when introducing education categories. These may be particularly confusing to non-Finish readers.

**Response**: We have now clarified this in the text and refer to the supplement, as requested (survey items).
